# A mouse model for triple-negative breast cancer tumor-initiating cells (TNBC-TICs) exhibits similar aggressive phenotype to the human disease

**DOI:** 10.1186/1471-2407-12-120

**Published:** 2012-03-27

**Authors:** Punit Kaur, Ganachari M Nagaraja, Hongying Zheng, Dawit Gizachew, Moses Galukande, Sunil Krishnan, Alexzander Asea

**Affiliations:** 1 Department of Pathology, Scott & White Memorial Hospital and Clinic and the Texas A&M Health Science Center, College of Medicine, Temple, TX 76504, USA; 2 Department of Medicine, Scott & White Memorial Hospital and Clinic and the Texas A&M Health Science Center, College of Medicine, Temple, TX 76508, USA; 3 Department of Surgery, Makerere University, P. O. Box 7072, Kampala, Uganda; 4 Department of Radiation Oncology, MD Anderson Cancer Center, 1515 Holcombe Boulevard, Houston, TX, USA; 5 Division of Investigative Pathology, Scott & White Memorial Hospital and Clinic and The Texas A&M Health Science Center College of Medicine, 1901 South 1st Street, Building 205, Temple, TX 76504, USA

**Keywords:** Triple-negative breast cancer, Mouse and human HspB1, Hsp25, Hsp27, Hsp72/HspA1A, Heat shock, Cancer stem cells, Tumor-initiating cells

## Abstract

**Background:**

Triple-negative breast cancer (TNBC) exhibit characteristics quite distinct from other kinds of breast cancer, presenting as an aggressive disease--recurring and metastasizing more often than other kinds of breast cancer, without tumor-specific treatment options and accounts for 15% of all types of breast cancer with higher percentages in premenopausal African-American and Hispanic women. The reason for this aggressive phenotype is currently the focus of intensive research. However, progress is hampered by the lack of suitable TNBC cell model systems.

**Methods:**

To understand the mechanistic basis for the aggressiveness of TNBC, we produced a stable TNBC cell line by sorting for 4T1 cells that do not express the estrogen receptor (ER), progesterone receptor (PgR) or the gene for human epidermal growth factor receptor 2 (HER2). As a control, we produced a stable triple-positive breast cancer (TPBC) cell line by transfecting 4T1 cells with rat HER2, ER and PgR genes and sorted for cells with high expression of ER and PgR by flow cytometry and high expression of the HER2 gene by Western blot analysis.

**Results:**

We isolated tumor-initiating cells (TICs) by sorting for CD24^+^/CD44^high^/ALDH1^+ ^cells from TNBC (TNBC-TICs) and TPBC (TPBC-TICs) stable cell lines. Limiting dilution transplantation experiments revealed that CD24^+^/CD44^high^/ALDH1^+ ^cells derived from TNBC (TNBC-TICs) and TPBC (TPBC-TICs) were significantly more effective at repopulating the mammary glands of naïve female BALB/c mice than CD24^-^/CD44^-^/ALDH1^- ^cells. Implantation of the TNBC-TICs resulted in significantly larger tumors, which metastasized to the lungs to a significantly greater extent than TNBC, TPBC-TICs, TPBC or parental 4T1 cells. We further demonstrated that the increased aggressiveness of TNBC-TICs correlates with the presence of high levels of mouse twenty-five kDa heat shock protein (Hsp25/mouse HspB1) and seventy-two kDa heat shock protein (Hsp72/HspA1A).

**Conclusions:**

Taken together, we have developed a TNBC-TICs model system based on the 4T1 cells which is a very useful metastasis model with the advantage of being able to be transplanted into immune competent recipients. Our data demonstrates that the TNBC-TICs model system could be a useful tool for studies on the pathogenesis and therapeutic treatment for TNBC.

## Background

Triple-negative breast cancer (TNBC) is characterized by a lack of receptor expression of estrogen receptor (ER) and progesterone receptor (PgR) and lack of gene expression for human epidermal growth factor receptor 2 (HER2) [1,2]. Chemotherapy remains the only possible therapeutic option in the adjuvant or metastatic setting in the TNBC. Importantly, the prognostic effect of TNBC is independent of poor grade, nodal status, tumor size and treatment [3]. The aggressiveness of TNBC is further indicated by the fact that: (i) the peak risk of recurrence occurs within the first 3 years after initial treatment of the disease with the majority of deaths occurring in the first 5 years [4] and (ii) after diagnosis of metastatic disease, a significantly shorter survival is observed in TNBC [4]. Conversely, the risk for late recurrences (i.e. beyond 5 years of diagnosis) is decreased by 50% compared with HER2-positive disease [5]. More than 90% of TNBC exhibit an invasive ductal histology and high histological grade, present with high mitotic index and carry central necrotic zones and pushing borders as well as a conspicuous lymphocytic infiltrate.

TNBC are typically observed in young African-American women, Hispanic women and Caucasian women who carry a mutation in the breast cancer 1 (BRCA1) early onset gene [6]. While some TNBC respond to chemotherapy, subsets of TNBC are chemotherapy-resistant and highly metastatic carrying with it an extremely poor prognosis [6]. The molecular mechanism, which governs the aggressive behavior of this subset of TNBC, is a matter of intense speculation, particularly since TNBC frequently express markers of epithelial mesenchymal transition (EMT). EMT is a normal developmental process in which cells of epithelial origin lose epithelial characteristics and polarity and acquire a mesenchymal phenotype associated with increased migratory behavior [7,8].

Self-renewing cancer cells are reported to be the only cell types within a tumor with an unlimited ability to initiate tumor growth and are therefore known as tumor-initiating cells (TICs), cancer stem cells or tumor-propagating cells [9-11]. The majority of carcinoma cells express a CD44^low^/CD24^high ^phenotype; however, a small subpopulation of carcinoma cells present within human breast cancers that exhibit a CD44^high^/CD24^low ^antigenic phenotype are highly enriched for TICs [12]. TNBC have been reported to have a higher percentage of CD44^high^/CD24^low ^expressing cells than other breast cancer subtypes [13].

The mouse twenty-five kDa heat shock protein (Hsp25/mouse HspB1) belongs to the family of small HSP and is the murine homologue of human twenty-seven kDa heat shock protein (Hsp27/human HspB1) which operates through ATP-independent mechanisms [14,15]. Elevated human HspB1 levels have been found in various tumors, including breast, prostate, gastric, uterine, ovarian, head and neck, and tumors arising from the nervous system and urinary system. Elevated levels of human HspB1 in ER-α positive benign neoplasia have been shown to promote the progression to more malignant phenotypes [16], increased anchorage independent tumor growth [17], increased resistance to chemotherapeutic drugs (including cisplatin and doxorubicin) and increased metastatic potential *in vitro *[18-20]. Furthermore, elevated human HspB1 expression in tumors correlated with shorter disease-free survival and recurrence in node-negative breast cancer [21,22], whereas its induction following chemotherapy also predicted poor prognosis and shorter disease-free survival [23].

Surface bound/expressed and total Hsp25 (mouse HspB1) and Hsp72 (HspA1A) on 4T1 murine breast adenocarcinoma tumors suggest that tumor development and metastatic spread favors cells that express high levels of mouse HspB1 on their plasma surface. However, tumors that express Hsp72/HspA1A on their plasma surface are sensitive to anti-tumor effector cells, which indicate increased surface expression of Hsp72/HspA1A on tumors and down regulation of human HspB1 expression could inhibit tumor growth and eliminate metastasis [24,25]. Enhanced expression of intracellular Hsp72/HspA1A has shown to be anti-inflammatory [26], anti-apoptotic [18], induce cell cycle arrest [27] and protect cells from stressful stimuli [28]. In contrast, enhanced extracellular expression of Hsp72/HspA1A either on the surface of tumors or in the extracellular milieu either enhances natural killer (NK) cell-mediated lysis [29] or upregulates antigen presenting cells (APC)-mediated acute phase responses [30-33], respectively. A study in which the mouse *hspb1 *gene has been silenced using interference ribonucleic acid (RNAi) technology suggests that mouse HspB1 has a profound effect on tumor proliferation and migration [24,25].

In this study, we constructed TNBC and TPBC by the stable transfection of parental 4T1 cells with the rat HER2, ER and PgR genes and sorted for cells with high expression of HER2, ER and PgR. We further produced TNBC-TICs and TPBC-TICs by sorting CD24^+^/CD44^high^/aldehyde dehydrogenase 1 (ALDH1)^+ ^expressing cells from TNBC and TPBC. Functional analysis demonstrated that TNBC-TICs exhibits a more aggressive phenotype than TNBC, TPBC, TPBC-TICs or parental 4T1 cells. Taken together, our studies suggest that these cells could be important preclinical model to characterize and effectively target cancer stem cells or TICs, and to further our understanding of the aggressive nature of TNBC found in humans.

## Methods

### Cells and culture conditions

4T1, a highly metastatic breast cancer cell line derived from a spontaneously arising BALB/c mammary tumor were purchased from American Type Culture Collection (ATCC; Rockville, MD). 4T1 cells were maintained in monolayer cultures in Dulbecco's modified Eagle's medium (DMEM) (Sigma Chemical, St. Louis, MO) supplemented with 10% fetal bovine serum (FBS). Cells were maintained at 37°C humidified atmosphere with 5% CO_2_.

### Animals and tumor challenge

Female BALB/c (H2^d^) wild type mice (6-8 weeks old) were purchased from Charles River Laboratories (Wilmington, MA) and housed under pathogen-free conditions in laminar flow isolation units in the Scott & White Hospital vivarium under alternate dark and light cycles, and maintained on food and water ad libitum. Female BALB/c (6-8 weeks old) were lightly anesthetized and the fur was shaved over the lateral thorax and a 5-mm-long incision was made to reveal mammary fat pad number 2. A 0.1-ml sample containing 10^4 ^tumors was injected into the mammary fat pad and the incision was closed with a wound clip. Tumor volume was measured once a week using an electronic caliper. The tumor volume was estimated using the formula of an ellipsoid (length × width × height × 0.5236). All animals were treated humanely and in accordance with the guidelines of the Committee on the Care and Use of Laboratory Animals of the Institute of Animal Resources, National Research Council and Institutional Animal Care and Use Committee (IACUC) of Scott & White Hospital. For each group 5 mice were used for statistical significance.

### Establishment of stable cell line

For the construction of HER^+^/ER^+^/PgR^+ ^4T1 cells (TPBC) the 550 bp rat human epidermal growth factor receptor 2 (HER2) (extracellular domain containing the herceptin domain) upstream primer (5'-GTCGAAGCTTATGGAGCTGGCGGCCTGG-3') and downstream primer (5'-GACTGAATTCTTAGTTGATGGGGCACGG-3'), and the 311 bp rat estrogen receptor (ER) upstream primer (5'-TGACTCTGCAGCAACAGCAT-3') and downstream primer (5'-GAGTTCTCAGATGGTGTTGG-3') and the 460 bp rat progesterone receptor (PgR) upstream primer (5'-AACTGGTTCCGCCACTCAT-3') and downstream primer (5'-AACTGGTTCCGCCACTCAT-3') were used and cloned into a pcDNA6.2/EmGFP construct according to the manufactures' instructions (Invitrogen). For stable transfection pcDNA6.2/EmGFP construct was linearized before transfection into 4T1 cells using Lipofectamine™ 2000 Reagent (Invitrogen). Similarly, for the construction of HER^-^/ER^-^/PgR^- ^4T1 cells (TNBC), the pcDNA6.2/EmGFP vector alone was used. Forty-eight hours after transfection, cells were split and growth media containing DMEM supplemented with 10% FBS and 10 μg/ml Blasticidin (Invitrogen) was added. After 4 days Blasticidin-resistant colonies were identified and further propagated. Cells stably transfected with enhanced green fluorescent protein (eGFP) were further enriched for eGFP-positive populations by sorting using a BD FACSAria flow cytometer (BD Biosciences) equipped with a 488 nm argon laser. The emission filter for eGFP was set at 515-545 nm. The presence of HER2, PgR and ER in TPBC was confirmed by Western blot analysis and PCR. Pure TNBC cells were obtained after Flow Cytometry sorting for ER^-^/PgR^- ^4T1 cells.

### Limiting dilution transplantation assay

After FACS sorting for 4T1wt, TNBC, TNBC-TICs, TPBC, TPBC-TICs expressing high levels of enhanced green fluorescence protein (eGFP), cells were washed once with ice-cold PBS and mixed in DMEM supplemented with 25% Matrigel (BD Biosciences). Female BALB/c (6-8 weeks old) were lightly anesthetized and the fur was shaved over the lateral thorax and a 5-mm-long incision was made to reveal mammary fat pad number 2. A 0.1-ml sample containing various concentrations of eGFP-bearing cells (500, 100, 50, 25 and 10) was injected into the mammary fat pad and the incision was closed with a wound clip. Confidence interval (95%) for repopulating mammary cell frequency were calculated using ELDA, a software application for limiting dilution analysis (LDA) as previously described [34]. All animals were treated humanely and in accordance with the guidelines of the Committee on the Care and Use of Laboratory Animals of the Institute of Animal Resources, National Research Council and Institutional Animal Care and Use Committee (IACUC) of Scott & White Hospital. For each group 5 mice were used for statistical significance.

### Live animal imaging

Live animal imaging was achieved by measuring the spectral fluorescence images captured using the Maestro™ *in vivo *imaging system (CRI, Woburn, MA). An excitation band pass filter from 445 to 490 nm and an emission filter over 515 nm were used. The tunable filter was automatically spaced in 10 nm increments from 500 to 720 nm while the camera captured fluorescence images at each wavelength with constant exposure. RGB (red-green-blue) color fluorescence images were synthesized from the spectral cube by mapping the spectral data into those color channels. All the fluorescence images obtained as RGB images were derived from the spectral datasets. Spectral unmixing was performed to segregate skin and hair auto fluorescence and to measure the true eGFP signal.

### Clonogenicity assay

The ability of tumor cells to metastasize to distant organs was assessed using the clonogenicity assay as previously described [26,35]. Briefly, mice were sacrificed and lung tissue was aseptically removed, minced with trypsin, seeded in triplicate at 1,000 cells/per 60 mm^3 ^petri-dish and incubated at 37°C in a 5% CO_2 _air atmosphere. Ten to twelve days later, the plates were washed twice with PBS and colonies were stained with crystal violet and counted. The colonizing efficiency was scored and results were compared to those in vehicle-treated cells.

### Protein separation and western blot analysis

Following various treatment protocols cells were washed once with complete medium, centrifuged and pellets lysed with 100 μl of lysing buffer containing a cocktail of protease inhibitors (antipain, bestain, chymostatin, E-64, pepstatin, phosphoramidon, pefabloc, ethylenediaminetetraacetic acid (EDTA), aprotinin; Complete Protease Inhibitor Cocktail Tablets^®^, Roche Diagnostics). Cells were then incubated for 30 min on ice and sonicated (Brandson 1510) for 15 min. The cell suspension was passed through a 26-gauge needle and protein quantification was performed using the Bradford method. Proteins were separated in a 10% sodium dodecyl sulfate polyacrylamide gel electrophoresis (SDS-PAGE) by carefully placing 30 μg of protein in each lane. Polyvinylidene fluoride (PVDF) (Millipore) was used to transfer the proteins and the membrane blocked with 5% skim milk (in TBS 1% pH 7.4 and 0.01% Tween 20) and incubated for 1 h at room temperature with appropriate primary antibodies; anti-Hsp72/HspA1A and anti-Hsp73/HspA8 (StressGen Biotechnologies, BC, Canada), or β-actin (Oncogene, San Diego, CA) with 1:1000 dilution. Blots were incubated 50 min at room temperature with 0.5 μg of appropriate species matched anti-peroxidase and the reaction was detected using the Luminol reagent for chemiluminescence (Santa Cruz Biotechnology). The intensity of the bands were analyzed by densitometry with a video densitometer (Chemilmager™ 5500; Alpha Innotech, San Leandro, CA) using the AAB software (American Applied Biology).

### Flow cytometry and cell sorting

For the isolation of TICs from TNBC and TPBC; 10^7 ^cells were stained with anti-CD24-FITC (1:100, BD Transduction Labs), anti-CD44-PE (1:200, BD Transduction Labs) and anti-ALDH1-Alexa-Fluor-568 (1:100, Molecular Probes) under previously optimized conditions. Isotype controls (IgG) were used as negative controls. CD24^+^/CD44^high^/ALDH1^+ ^and CD24^-^/CD44^-^/ALDH1^- ^cells were sorted using a BD FACSort with a Lysys II software program (BD Biosciences). Individual cells were gated based on forward (FSC) and orthogonal scatter (SSC). Cell debris was excluded by raising the FSC-height photomultiplier (PMT) threshold. The PMT for FITC (FL1-height), PE (FL2-height) and Alexa-Fluor 568 (FL-3-height) was set on a logarithmic scale. The flow rate was adjusted to < 200 cells/second. Post sorted cells were collected in cell culture medium containing 20% FBS and then washed and returned to 4T1 complete medium; DMEM supplemented with 10% FBS and maintained at 37°C humidified atmosphere with 5% CO_2_.

### Fluorescence microscopy

Standard fluorescence microscopy was performed using an Olympus CKX41 microscope. A DP71 CCD camera was used to capture phase contrast and eGFP fluorescence images with DP71 image acquisition interface software (Olympus).

### Statistical analysis

For comparisons between groups, Dunn multiple comparison tests and student *t*-test and one-way analysis of variance (ANOVA) were used in this study (p values < 0.001 were considered significant).

## Results

### Development of mouse TNBC, TPBC and breast cancer Tumor-Initiating Cells (TICs)

**Figure 1:**
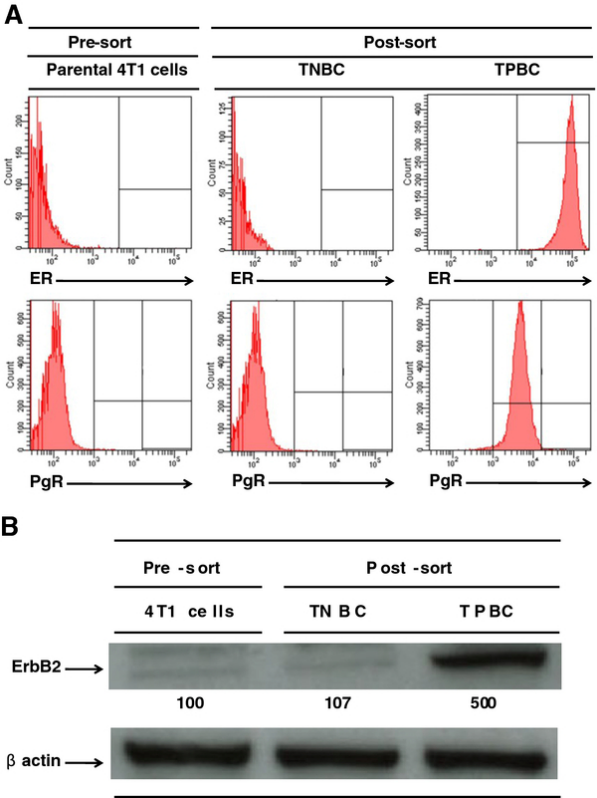
**Transfection of rat HER2, ER and PgR genes into 4T1 cells produces a population of TNBC and TPBC**. (A) Parental 4T1 cells (10^6^), TNBC (10^6^), or TPBC (10^6^) were placed on ice and stained with anti-ER-FITC (top panels) or anti-PgR-PE (bottom panels). The surface intensity for these antibodies were analyzed by flow cytometry using a FACSort with a Lysys II software program (Becton & Dickinson). Individual cells were gated based on forward (FSC) and orthogonal scatter (SSC). Cell debris was excluded by raising the FSC-height PMT threshold. Histograms are mean relative counts and are a representative experiment of three independently performed experiments with similar results. (B) Parental 4T1 cells (10^6^) (lane 1), TNBC (10^6^) (lane 2), or TPBC (10^6^) (lane 3) were lysed and the amount of ErbB2 (HER2) was determined by Western blot analysis using specific Mab as described in detail in the Materials and Methods section. β-actin was used as control for equal loading. The intensity of the bands were analyzed by densitometry with a video densitometer (Chemilmager™ 5500; Alpha Innotech, San Leandro, CA) using the AAB software (American Applied Biology). Data is a representative experiment from three independently performed experiments with similar results.

To understand the molecular characteristics of TNBC, and determine the reason for its highly aggressive phenotype as compared to TPBC, we used the 4T1 breast adenocarcinoma mouse cell line as a model system, because tumor growth and metastatic spread of 4T1 cells very closely mimic human breast cancer and has the advantage of being able to be transplanted into immune competent recipients. To produce a population of TNBC cells, we transfected parental 4T1 cells with empty vector control and sorted for cells that do not express HER2, ER or PgR (HER2^-^/ER^-^/PgR^-^) using flow cytometry and sorted to > 90% purity termed TNBC (Figure 1A; middle panels). Western blot analysis revealed that there was no significant increase in the HER2 expression in TNBC, as compared to parental 4T1 cells and no expression of either ER or PgR (Figure 1B; lane 2). We then constructed a population of 4T1 breast cancer cells to mimic the TPBC phenotype in humans, and that are phenotypically HER2^+^/ER^+^/PgR^+^. The rationale for creating a TPBC 4T1 cell line is to act as a relevant control for the TNBC 4T1 cell line. This was achieved by stable transfection of parental 4T1 cells (Figure 1A; left panels) with the rat HER2, ER and PgR genes to avoid non-specific immune responses. Cells were then sorted for high expression of ER and PgR by flow cytometry to > 90% purity and termed TPBC (Figure 1A; right panels). Western blot analysis revealed that the expression of HER2 (using anti-ErbB2 antibodies) in TPBC increased approximately 500% above parental 4T1 cell levels (Figure 1B; lane 3).

**Figure 2:**
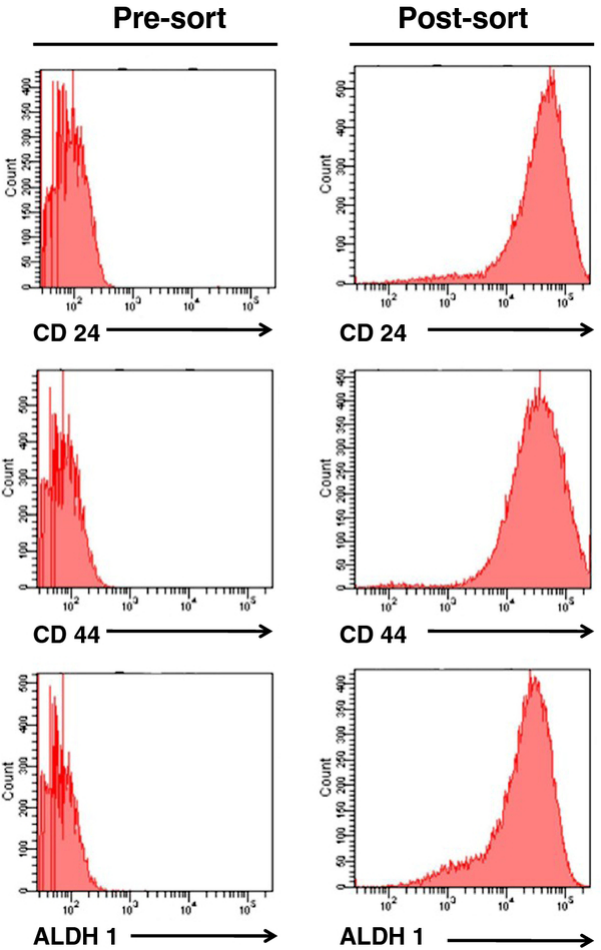
**Selection of triple-negative breast cancer-cancer stem cells (TNBC-TICs)**. CD24, CD44 and ALDH1 antibodies were used to isolate a population of breast cancer tumor-initiating cells (TICs) from TNBC. TNBC (10^6^) were placed on ice and stained with anti-CD24-FITC (top panels), anti-CD44-PE (middle panels) and anti-ALDH1 (bottom panels). Cells were sorted using a FACSort with a Lysys II software program (Becton & Dickinson). Individual cells were gated based on forward (FSC) and orthogonal scatter (SSC). Cell debris was excluded by raising the FSC-height PMT threshold. Histograms are mean relative counts and are a representative experiment of three independently performed experiments with similar results.

To isolate a population of breast cancer tumor-initiating cells (TICs), we stained the TNBC (HER2^-^/ER^-^/PgR^-^) and TPBC (HER2^+^/ER^+^/PgR^+^) described above with antibodies known to differentiate breast cancer stem cells, including CD24 (a cell adhesion molecule), aldehyde dehydrogenase 1 (ALDH1; an enzyme that catalyses the oxidation of aldehydes) and CD44 (a cell-surface glycoprotein involved in cell-cell interactions, cell adhesion and migration). We uncovered two phenotypically distinct populations in each group; a population expressing CD24^+^/ALDH1^+^/CD44^high ^and another population expressing CD24^-^/ALDH1^-^/CD44^low ^cells (data not shown). Using flow cytometry sorting techniques, we sorted for CD24^+^/ALDH1^+^/CD44^high ^cells to a > 90% purity (Figure 2).

**Figure 3:**
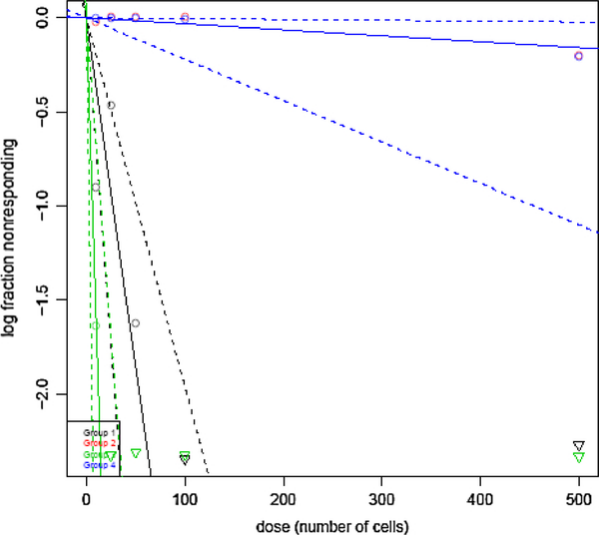
**CD24^+^/ALDH1^+^/CD44^high ^identifies a population of TICs in both TNBC and TPBC cell fractions**. The log-fraction plot of the limiting dilution model has been generated using ELDA software for limiting dilution analysis (LDA) after fitting the data from Table 1. The slopes for CD24^+^/ALDH1^+^/CD44^high ^cells derived from TNBC (green line), CD24^+^/ALDH1^-^/CD44^low ^cells derived from TNBC (blue line), CD24^+^/ALDH1^+^/CD44^high ^cells derived from TPBC (black line), CD24^+^/ALDH1^-^/CD44^low ^cells derived from TPBC (red line), is the log-active cell fraction. The dotted lines give the 95% confidence interval.

Limiting dilution transplantation experiments were then performed to validate that the CD24^+^/ALDH1^+^/CD44^high ^cells do in fact have greater tumor initiating ability than CD24^+^/ALDH1^-^/CD44^low ^cells. Our results demonstrated that CD24^+^/ALDH1^+^/CD44^high ^cell fraction derived from TNBC and TPBC contain cells with a significantly greater ability to repopulate the breast tissue, as compared to CD24^+^/ALDH1^-^/CD44^low ^cells derived from TNBC or TPBC (Table 1). The log-fraction plot of the limiting dilution model was generated using ELDA software for limiting dilution analysis (LDA) after fitting the data from Table 1. We demonstrated that the estimated 95% confidence intervals for repopulating mammary cells frequencies for CD24^+^/ALDH1^+^/CD44^high ^cells derived from TNBC and TPBC are 6.22 and 27.02, respectively, as compared to 3168.93 for CD24^+^/ALDH1^-^/CD44^low ^cells derived from TNBC and TPBC (Table 2). The overall test for differences in stem cell frequencies between any of the groups gave a Chi-Square score of 142 (p = 1.29 × 10^-30^). The likelihood ratio test, which is designed to test whether the single-hit model is correct gave a Chi-Square of 0.00411 (p = 0.949). The score test of heterogeneity, which is designed to test whether the different cultures (assays) have the same active cell proportion gave a Chi-Square of 0.728 (p = 0.394). The slopes for CD24^+^/ALDH1^+^/CD44^high ^cells derived from TNBC (green line) and TPBC (black lines), significantly differ from CD24^+^/ALDH1^-^/CD44^low ^cells derived from either TNBC (blue line) or TPBC (red line), which overlap due to similar values (Figure 3). The estimated slope value is 1.02, since the value is greater than 1, this suggests a multi-hit model in which two or more cells are synergistically required to produce a positive response (Figure 3). We therefore termed the CD24^+^/ALDH1^+^/CD44^high ^cells tumor-initiating cells (TICs). Thus, TICs derived from TNBC were designated TNBC-TICs and TICs derived from TPBC were designated TPBC-TICs.

**Table 1:** TNBC-TICs exhibit a greater clonogenic growth potential as compared to TNBC, TPBC-TICs, TPBC or parental 4T1 cells

Cell type^1^	Number of cells transplanted	Fraction of mice with tumors^2^
TPBC-derived:		
CD24^+^/ALDH1^+^/CD44^high^	500	5/5
CD24^+^/ALDH1^+^/CD44^high^	100	5/5
CD24^+^/ALDH1^+^/CD44^high^	50	4/5
CD24^+^/ALDH1^+^/CD44^high^	25	2/5
CD24^+^/ALDH1^+^/CD44^high^	10	3/5
TPBC-derived:		
CD24^+^/ALDH1^-^/CD44^low^	500	1/5
CD24^+^/ALDH1^-^/CD44^low^	100	0/5
CD24^+^/ALDH1^-^/CD44^low^	50	0/5
CD24^+^/ALDH1^-^/CD44^low^	25	0/5
CD24^+^/ALDH1^-^/CD44^low^	10	0/5
TNBC-derived:		
CD24^+^/ALDH1^+^/CD44^high^	500	5/5
CD24^+^/ALDH1^+^/CD44^high^	100	5/5
CD24^+^/ALDH1^+^/CD44^high^	50	5/5
CD24^+^/ALDH1^+^/CD44^high^	25	5/5
CD24^+^/ALDH1^+^/CD44^high^	10	4/5
TNBC-derived:		
CD24^+^/ALDH1^-^/CD44^low^	500	1/5
CD24^+^/ALDH1^-^/CD44^low^	100	0/5
CD24^+^/ALDH1^-^/CD44^low^	50	0/5
CD24^+^/ALDH1^-^/CD44^low^	25	0/5
CD24^+^/ALDH1^-^/CD44^low^	10	0/5

^1^Tumor cells derived from TPBC or TNBC cells were stably transfected with eGFP using the lentivirus transduction technique as described in detail in the Methods section

^2^Twenty-one days post tumor cell transplantation into the mammary pad of naïve female BALB/c mice, animals were scarified and eGFP signal from the tumors were measured using the Maestro™ *in vivo *imaging system (CRI), and spectral unmixing was performed to segregate skin and hair auto fluorescence and to measure the true eGFP signal, as described in detail in the Methods section. Data is the fraction of mice with tumors (n = 5)

**Table 2:** Confidence interval (95%) for repopulating mammary cell frequency

Cell type	Confidence intervals for 1/(TICs frequency)^1^		
	
	Lower	Estimate	Upper
TPBC-derived:			
CD24^+^/ALDH1^+^/CD44^high^	51.0	27.02	14.4
CD24^+^/ALDH1^-^/CD44^low^	22124.7	3168.93	454.3
TNBC-derived:			
CD24^+^/ALDH1^+^/CD44^high^	14.9	6.22	2.8
CD24^+^/ALDH1^-^/CD44^low^	22124.7	3168.93	454.3

^1^Confidence interval (95%) for repopulating mammary cell frequency for the data in Table 1. Data were generated using ELDA, a software application for limiting dilution analysis (LDA) as previously described [34]

### Mouse TNBC-TICs proliferates faster and metastasizes to a greater extent than TPBC-TICs or parental 4T1 wild type cells

**Figure 4:**
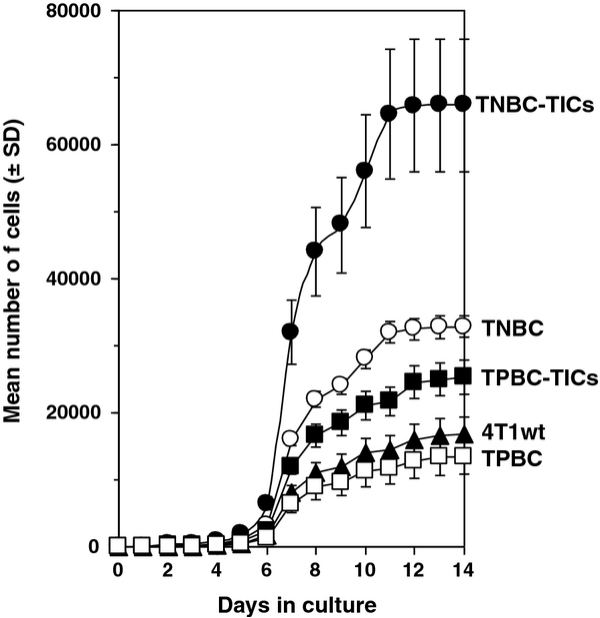
**TNBC-TICs proliferate significantly faster than TNBC, TPBC-TICs, TPBC or parental 4T1 cells**. TNBC-TICs (filled circles), TNBC (open circles), TPBC-TICs (filled squares), TPBC (open squares), or parental 4T1 cells (filled triangles) were seeded at 10^4 ^cells into T-250 tissue culture flasks on day 0 in media containing DMEM supplemented with 10% FBS. At various times cell count was determined using a hemocytometer under a phase-contrast light microscope. Data represent the mean number of cells ± SD and is the sum of four independently performed experiments performed in quadruplicates.

**Figure 5:**
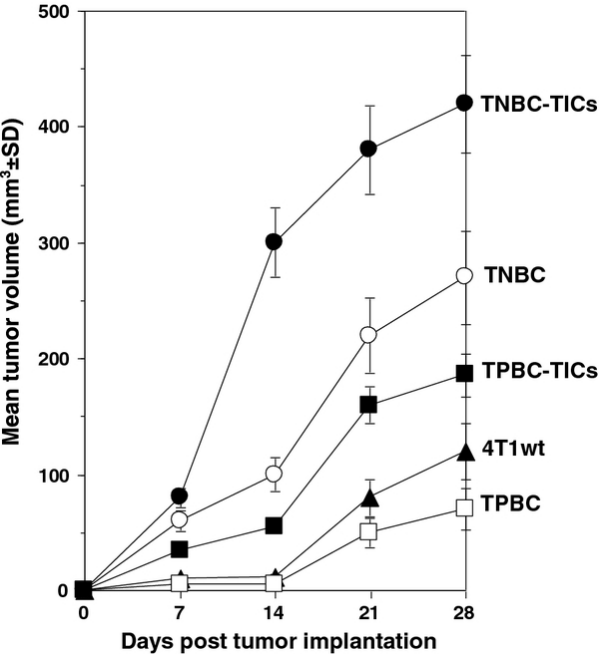
**Tumors from TNBC-TICs grow significantly larger than TNBC, TPBC-TICs, TPBC or parental 4T1 cells**. Implantation of TNBC-TICs into female BALB/c mice resulted in larger tumors than TNBC, TPBC-TICs, TPBC or parental 4T1 cells. TNBC-TICs (filled circles), TNBC (open circles), TPBC-TICs (filled squares), TPBC (open squares), or parental 4T1 cells (filled triangles) 10^4 ^cells were implanted into the left breast pad of female BALB/c mice (6-8 weeks old) and tumor growth was measured using an electronic calipher. Data are mean tumor volume mm^3 ^± SD and are the sum of three independently performed experiments with five mice per group.

**Figure 6:**
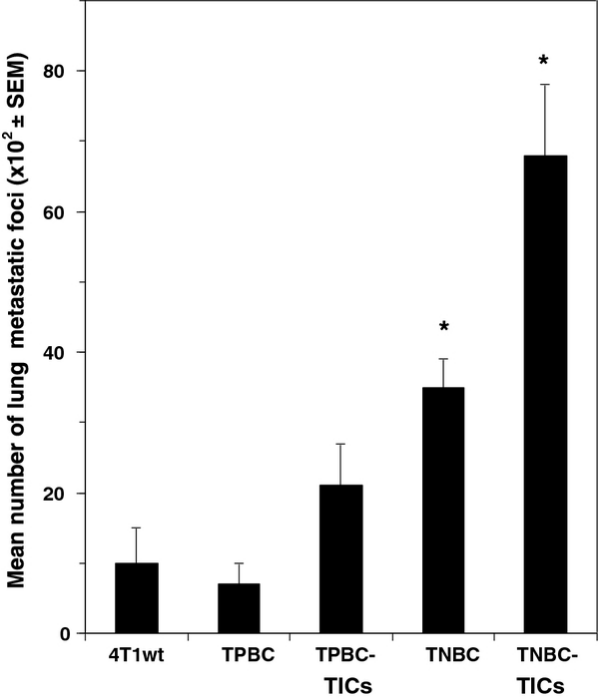
**TNBC-TICs produce significantly more metastatic foci than TNBC, TPBC-TICs, TPBC or parental 4T1 cells**. Female BALB/c mice (6-8 weeks old) were injected with 10^4 ^parental 4T1 cells (4T1wt), triangles), TPBC, TPBC-TICs, TNBC or TNBC-TICs into the left breast pad. Twenty-one days post tumor cell implantation animals were euthanized and lungs filled with a solution of India Ink (1:6 in PBS) *via *intratracheal route. Bars represent the mean number of lung metastatic foci (10^2 ^× SEM) and are the sum of three independently performed experiments with five mice per group. *p < 0.001 vs parental 4T1 cells (4T1wt).

We further demonstrated that CD24^+^/ALDH1^+^/CD44^high ^cells derived from TNBC (TNBC-TICs) proliferated approximately 7-fold more than parental 4T1 cells, and that TPBC-TICs and TNBC proliferated 2-fold and 3-fold more than the parental 4T1 cells, respectively. We demonstrated that TPBC did not proliferated significantly more than the parental 4T1 cells *in vitro *(Figure 4). To determine the clonogenic growth potential and migratory properties of the newly developed TICs, we implanted TICs into the breast pad of naïve female BALB/c mice and demonstrated that TNBC-TICs developed tumors approximately 4-fold larger than parental 4T1 cells (Figure 5). We further demonstrated that TPBC and TNBC produced tumors 1.5-fold and 2.5-fold larger than tumors developed by the parental 4T1 cells (Figure 5). To negate the possibility that hematoxylin and eosin (H&E) staining of lung tissues might inadvertently miss micro-metastasis, we used the clonogenicity assay on the lungs of isolated 21 days post tumor cell implantation and revealed that TNBC-TICs and TNBC developed approximately 5-fold and 2-fold more metastatic foci than the parental 4T1 cells, respectively (data not shown). Interestingly, TPBC-TICs and TPBC did not produce significantly more metastatic foci than the parental 4T1 cells (Figure 6).

**Figure 7:**
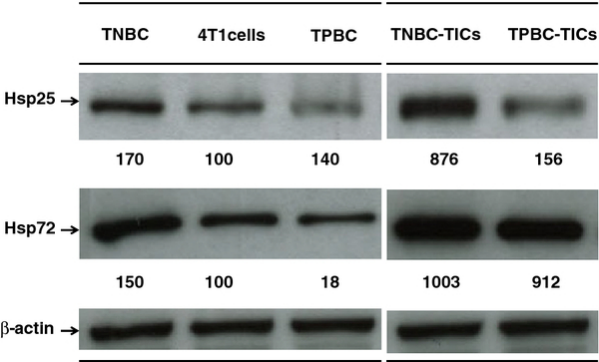
**TNBC-TICs express higher levels of Hsp25 and Hsp72/HspA1A than TNBC, TPBC-TICs, TPBC or parental 4T1 cells**. TNBC (10^6^) (lane 1), parental 4T1 cells (10^6^) (lane 2), TPBC (10^6^) (lane 3), TNBC-TICs (10^6^) (lane 4) or TPBC-TICs (10^6^) (lane 5) were lysed and the amount of Hsp25 or inducible Hsp72/HspA1A was determined by Western blot analysis using specific Mab as described in detail in the Materials and Methods section. β-actin was used as control for equal loading. The intensity of the bands were analyzed by densitometry with a video densitometer (Chemilmager™ 5500; Alpha Innotech, San Leandro, CA) using the AAB software (American Applied Biology). Data is a representative experiment from three independently performed experiments with similar results.

High levels of mouse HspB1 have been shown to be associated with increased tumor growth and high metastatic potential [24,25,36]. To determine if the increased metastatic potential exhibited by TNBC-TICs is associated with increased mouse HspB1expression, we probed the cells with mouse HspB1 and Hsp72/HspA1A after Western blot analysis and demonstrated that TNBC-TICs express 876% higher levels of mouse HspB1 1,003% higher levels of Hsp72/HspA1A as compared to parental 4T1 cells (Figure 7).

## Discussion

This study was undertaken to address a critical need in TNBC research; develop a model system which can be used to elucidate the reason for the apparent highly aggressive phenotype of triple-negative breast cancer (TNBC). We have successfully developed four cell lines derived from the parental 4T1 breast adenocarcinoma mouse cell lines that can be used to study the pathogenesis and therapeutic treatment for TNBC.

Limiting dilution transplantation experiments validate that CD24^+^/ALDH1^+^/CD44^high ^cells have a significantly greater tumor initiating ability than CD24^+^/ALDH1^-^/CD44^low ^cells (Tables 1 and 2). In addition, 95% confidence intervals for repopulating mammary cells frequencies clearly demonstrated that CD24^+^/ALDH1^+^/CD44^high ^cells derived from TNBC and TPBC have a greater repopulating ability than CD24^+^/ALDH1^-^/CD44^low ^cells derived from TNBC and TPBC (Table 2), and is confirmed by log-fraction plot of the limiting dilution model has been generated using ELDA software (Figure 3). Interestingly, CD24^+^/ALDH1^+^/CD44^high ^cells derived from TNBC (TNBC-TICs) were approximately 4-fold more efficient than CD24^+^/ALDH1^+^/CD44^high ^cells derived from TPBC (TPBC-TICs) (Table 2). The exact reason for this difference is currently under investigation in our laboratory (Kaur et al., *in preparation*). However, the data showing that TNBC-TICs exhibit classical signs of aggressive phenotype shown by human TNBC including increased tumor growth (Figure 5) and enhanced metastatic potential (Figure 6), led us to hypothesize that the differential expression of mouse HspB1 and Hsp72/HspA1A, might be a possible explanation (Figure 7). We demonstrated that TNBC-TICs express significantly higher levels of mouse HspB1 and Hsp72/HspA1A than TNBC, TPBC, TPBC-TICs or parental 4T1 cells (Figure 7). This data is in agreement with previous findings that elevated levels of mouse and human HspB1 promotes the progression of tumor to more malignant phenotypes [16,24,25,37], and that elevated human HspB1 expression in tumors correlated with shorter disease free survival and recurrence in node-negative breast cancer [21,22]. Our previous studies demonstrated that selection of 4T1 cells based on the expression of mouse HspB1 selects a particular phenotype with increased metastatic potential [25]. Further studies have demonstrated that increased metastatic potential of 4T1 cells expressing high levels of mouse HspB1 can be abrogated by suppressing the expression of mouse HspB1 using small interfering RNA (siRNA) directed against the mouse *hspb1 *gene [24]. Nagaraja et al. recently reported that tumors with high levels of HspB1 are able to evade the immune system and rapidly metastasize, in part, due to HspB1 acting as a repressor of proteasome function, which in turn results in insufficient presentation of tumor-associated antigens on the surface of tumors and a lack of recognition by CD8^+ ^cytotoxic T lymphocytes (CTL) [36]. These authors demonstrated that short term silencing of HspB1 using siRNA or permanent silencing using lentivirus-RNAi technology enhanced proteasome activity, abrogated metastatic potential, induced the regression of established breast tumors by tumor-specific CD8^+ ^T cells and stimulated long-lasting memory responses [36].

Our study suggests that CD24^+^/ALDH1^+^/CD44^high ^identifies TICs in both TNBC and TPBC cell fractions, TNBC-TICs and TPBC-TICs respectively. These cells clearly exhibit ability to aggressively repopulate the mammary tissue of mice in limiting dilution transplantation assays (Tables 1, 2 and Figure 3). The expression of aldehyde dehydrogenase 1 (ALDH1) a marker associated with cancer stem cell-like or TICs features, was significantly more common in ER^- ^tumors. ALDH1^+ ^cells were the least frequent in luminal A and B tumors compared to basal-like and HER2^+ ^subtypes, correlating with a recent study in breast cancer cell lines [38]. CD44^+^/CD24^- ^cells and ALDH1^+ ^cells were more frequently found in the basal-like than in luminal subtypes, however, CD44^+^/CD24^- ^cells but not ALDH1^+ ^cells were less common in HER2^+ ^than in basal-like cases. In normal breast tissue, CD44^+^/CD24^- ^cells were limited to the basal layer whereas ALDH1^+ ^cells were found in both basal and luminal compartments suggesting that ALDH might be a marker of both bipotential mammary epithelial stem cells and luminal lineage committed progenitors. The difference in the relative frequency of CD44^+^/CD24^- ^and ALDH1^+ ^cells between basal-like and HER2^+ ^tumor subtypes may reflect their distinct cell-of-origin or the alteration of stem cell-like gene expression programs due to tumor subtype-specific transforming events [39]. All markers identifying cancer stem cells are surface markers such as epithelial cell adhesion molecule (EpCAM), CD44, CD24, CD133 and C-X-C chemokine receptor type 4 (CXCR4) definitely expressed on the normal stem cells at the same time and apparently changing during cancer stem cell development. Rasheed et al. reported that ALDH could potentially be an endogenous marker for pancreatic TICs after experiments demonstrated significant clonogenic growth potential and migratory properties *in vitro *and *in vivo *[40]. In addition, these authors detected 90/269 ALDH^+ ^tumors in primary surgical specimens, and verified that their presence was associated with worse survival [40].

Previous studies clearly demonstrated that the high expression of human HspB1 in tumors correlates with increased tumor growth and enhanced metastatic potential [41]. In addition, the high surface expression of mouse HspB1 on 4T1 tumors significantly increases their ability to grow within the abdominal breast gland and to successfully metastasize and colonize the lungs and the interaction between host effector cells and tumors expressing high levels of surface-bound HspB1 results in abrogation and/or deactivation of host anti-tumor responses [25,37,41-43]. The rational for this study is that triple-negative breast cancer (TNBC) is as a type of breast cancer that has been described to be more aggressive than other kinds of breast cancer [4,44-48]. We hypothesized that a possible mechanism by which TNBC exhibit increased aggression and resistance to chemotherapy as compared to other cancers is, in part, due to the high expression of mouse and human HspB1.

## Conclusions

In conclusion, these studies suggest that these cell lines could provide an important preclinical model for the characterization and effective targeting of cancer stem cells or TICs, and to help further our understanding of the aggressive nature of TNBC found in humans. This is significant because TNBC although slightly responsive to chemotherapy are significantly more difficult to treat and generally insensitive to most available hormonal or targeted therapeutic agents than other kinds of breast cancer. The cell lines described in this study will allow for a better understanding of the reason for this aggressive nature and will pave the way for the design of more effective therapeutics.

## Abbreviations

ALDH1: Aldehyde dehydrogenase 1; BRACA1: Breast cancer 1 early onset; CSC: Cancer stem cells; CTL: Cytotoxic T lymphocytes; CXCR4: C-X-C chemokine receptor type 4; EMT: Epithelial mesenchymal transition; eGFP: Enhanced green fluorescence protein; EpCAM: Epithelial cell adhesion molecule; ER: Endoplasmic reticulum; PgR: Progesterone receptor; HER2: Human epidermal growth factor receptor 2; HSP: Heat shock proteins; *hsp*: Heat shock protein gene; Hsp25/HspB1: Mouse twenty-five kDa heat shock protein; Hsp27/HspB1: Human twenty-seven kDa heat shock protein; Hsp72/HspA1A: Seventy-two kDa heat shock protein; LDA: Limiting dilution analysis; MHC: Major histocompatibility complex; NK: Natural killer; RNAi: Interference ribonucleic acid; siRNA: Small interference ribonucleic acid; TICs: Tumor-initiating cells; TNBC: Triple-negative breast cancer; TPBC: Triple-positive breast cancer.

## Competing interests

The authors declare that they have no competing interests.

## Authors' contributions

AA conceived and designed the experiments. PK, GMN and HZ performed the experiments. PK, GMN, HZ, DG, MG, SK and AA analyzed the data. DG, MG, SK and AA contributed reagents/materials/analysis tools. PK and AA wrote the paper. All authors read and approved the final manuscript.

## Pre-publication history

The pre-publication history for this paper can be accessed here:

http://www.biomedcentral.com/1471-2407/12/120/prepub
